# Vaccinia virus egress mediated by virus protein A36 is reliant on the F12 protein

**DOI:** 10.1099/jgv.0.000816

**Published:** 2017-06-20

**Authors:** David C. J. Carpentier, Alexander Van Loggerenberg, Nele M. G. Dieckmann, Geoffrey L. Smith

**Affiliations:** Department of Pathology, University of Cambridge, Tennis Court Road, Cambridge CB2 1QP, UK; ^†^​ Present address: Cambridge Institute for Medical Research (CIMR), University of Cambridge, Cambridge Biomedical Campus, CB2 0XY, UK.

**Keywords:** vaccinia virus, virus egress and spread, proteins F12 and A36, kinesin-1, microtubule, actin nucleation

## Abstract

Egress of vaccinia virus from its host cell is mediated by the microtubule-associated motor kinesin-1, and three viral proteins, A36 and the F12/E2 complex, have been implicated in this process. Deletion of F12 expression causes a more severe reduction in egress than deletion of A36 but whether these proteins are involved in the same or different mechanisms of kinesin-1 recruitment is unknown. Here it is shown that a virus lacking both proteins forms a smaller plaque than mutants lacking either gene alone, indicating non-redundant functions. A36 not only links virions directly to kinesin-1 but also nucleates actin polymerization to propel surface virions away from the host cell. To address the relative importance of these functions for virus spread, a panel of recombinant viruses was constructed in which the ability of A36 to bind kinesin-1 or to nucleate actin polymerization was abrogated individually or together, in the presence or absence of F12 expression. Analysis of these viruses revealed that in the presence of the F12 protein, loss of kinesin-1 interaction made a greater contribution to plaque size than did the formation of actin tails. However in the absence of F12, the ability of A36 to promote egress was abrogated. Therefore, the ability of A36 to promote egress by kinesin-1 is reliant on the F12 protein.

## Abbreviations

CEV, cell-associated enveloped virus; EEV, extracellular enveloped virus; IEV, intracellular enveloped virus; IMV, intracellular mature virus; KLC, kinesin light chain; MT, microtubule; p.i., post-infection; VACV, vaccinia virus.

## Introduction

Vaccinia virus (VACV) is the prototypic member of the *Poxviridae,* a family of large, complex DNA viruses that replicate in the cytoplasm of host cells [1] and includes variola virus, the causative agent of smallpox [2]. VACV is a valuable model to study cytoskeleton-mediated trafficking because it hijacks both the microtubule (MT) and actin networks to facilitate virus transport within and between cells [3, 4]. Upon entry into a cell, VACV cores migrate into the cell interior in an MT-dependent manner [5] to form virus factories where new virions are assembled [6]. The first infectious virions formed are intracellular mature virus (IMV) or mature virus (MV) [7]. Some IMVs migrate away from viral factories in an MT-dependent process [8] and become wrapped by a double layer of early endosomal [9] or trans-Golgi [10] membranes, to form intracellular enveloped virus (IEV), also called wrapped virus (WV). IEVs are in turn transported towards the cell surface in an MT-dependent process [11–14] where their outer envelope fuses with the cell membrane, exposing the virion on the cell surface. Virions that remain attached to the host cell are known as cell-associated enveloped virus (CEV) and can induce a transmembrane signal that stimulates actin polymerization, resulting in formation of an actin tail propelling the virion away from the cell (reviewed in [3, 15]). Released virions are called extracellular enveloped virus (EEV) (reviewed in Roberts and Smith [4]). These virions mediate long range spread of virus in cell culture and *in vivo* [16], and are resistant to complement due to incorporation of host complement control proteins into the EEV envelope [17].

During IEV formation, virions acquire a double envelope containing at least five virus integral membrane proteins: B5 [18, 19], A33 [20], A34 [21], A36 [22, 23] and A56 [24]. In addition, protein F13 is attached to the membrane via acylated cysteine residues [25], and proteins F12 [26] and E2 [27] are indirectly and transiently associated with the IEV particle during egress [28]. All of these proteins, except A56, interact with at least one other member of this group [29] and are involved in the formation and/or egress of IEVs [30]. Of these, A36 [22, 31], F12 [26, 32] and E2 [27, 33] are involved in MT-mediated IEV egress.

IEV egress is mediated by kinesin-1 [14], also known as conventional kinesin, the prototype member of the kinesin protein superfamily [34]. Kinesin-1 is a tetrameric complex consisting of two copies of the kinesin heavy chain (KHC) and two copies of the kinesin light chain (KLC). A36 possesses two copies of a WE/D motif (a tryptophan residue followed by either a glutamic acid or aspartic acid residue) that form a bipartite kinesin-1 interaction motif [33] also found in cellular kinesin-interacting proteins [35, 36]. Peptides containing this WE/D motif interact with a binding groove formed by the tetratricopeptide repeat (TPR) cargo interaction region of KLC [37].

Unlike most of the other IEV envelope proteins, A36 is associated predominantly with the outer IEV envelope and after virion release it accumulates on the plasma membrane beneath CEVs [23]. Phosphorylation at tyrosine 112 and 132 by Src and Abl family kinases results in recruitment of Nck, Grb2, Cdc42, N-WASP and WIP, to nucleate actin polymerization via the Arp2/3 complex [38, 39], producing actin tails. A36 and A33 expressed early during virus replication form a complex on the cell surface and induce actin tails beneath superinfecting CEV/EEV virions that repel the virions away from the cell and enhance virus spread [40, 41].

F12 associates with IEVs in an A36-dependent manner [42], and indirectly with KLC via its binding partner E2 [43]. While both A36 and F12 have been implicated in IEV egress, and viruses lacking expression of either protein are severely attenuated *in vivo* [22, 26], their relative importance for IEV egress is different. Viruses lacking F12 expression show a more severe reduction in egress [26] than viruses lacking A36 expression [44]. It is unclear if A36 and F12 contribute to IEV egress together via the same process or independently via separate processes. The ability of IEVs to move at MT-dependent trafficking speeds in the absence of A36 [44] and the reported movement of IEVs in the absence of F12 [28] would suggest that two independent processes are involved.

To address this issue, a mutant virus was constructed lacking both proteins. This virus formed smaller plaques than viruses lacking either A36 or F12 alone. To determine the contribution of A36-mediated actin tail formation and A36-mediated kinesin-1 recruitment to this phenotype, a panel of viruses was constructed in which A36 was mutated to lose either or both of these properties. These mutations were introduced into viruses that do or do not express F12. Analysis of these viruses revealed that in the presence of F12 both properties of A36 contributed to spread. However, in the absence of F12, even when A36 retained the WE/D motifs required for interaction with KLC, A36 was unable to contribute to IEV egress.

## Results

### Deletion of A36 and F12 has a cumulative effect on virus spread but not on IEV egress

**Fig. 1.**
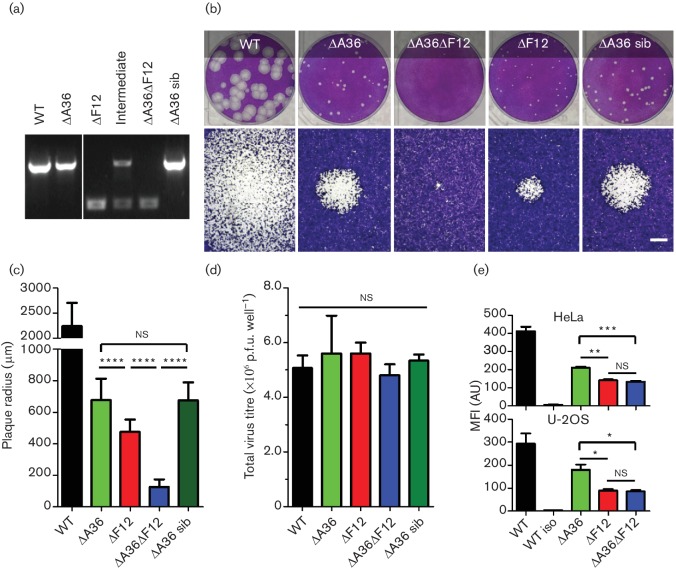
Deleting both A36 and F12 expression from VACV has a cumulative detrimental effect on spread but not egress. (a) PCR analysis of recombinant virus genomes to confirm the deletion of the F12 locus in the double deletion virus v∆A36∆F12-A5GFP. PCR fragments were generated using primers flanking the deleted region with an ~2 kb band indicating presence of the WT allele and a 500 bp band indicating the ∆F12 allele. Analysis of the intermediate virus possessing both alleles is included. (b) Plaque size comparison of vA5GFP (WT), v∆A36-A5GFP (∆A36), v∆F12-A5GFP (∆F12) and the newly generated v∆A36∆F12-A5GFP (∆A36∆F12) and its sibling (sib). Monolayers of BS-C-1 cells were infected with about 30 p.f.u. per well and stained 7 days post-infection (p.i.). A single 35 mm diameter well (top) is shown as well as a single plaque (bottom, scale bar represents 500 µm). (c) Quantification of plaque size analysis. The mean plaque diameter was calculated from measurements taken from a minimum of 45 individual plaques from three independently-infected wells (~15 measurements per well). Statistical significance calculated by Student’s t-test. ****, *P*<0.0001. (d) Analysis of virus replication. Viruses were used to infect a monolayer of BS-C-1 cells in a 35 mm diameter dish at 10 p.f.u. per cell. Virus-infected cells were harvested 24 h later and total infectious virus present in the medium and cells was titrated by plaque assay. Values represent means from three independent samples. None of the samples showed a statistically significant difference in titres when compared to the WT sample by Student's t-test, or when all the means were compared by one-way ANOVA. (e) Measurement of cell surface virus by flow cytometry. HeLa or U-2 OS cells were infected at 5 p.f.u. per cell and stained for surface B5 prior to fixation and analysis by flow cytometry. Mean fluorescence intensity (MFI) per cell is shown as the mean (in arbitrary units, AU) of three independently-infected samples (>2000 events analysed per sample). Statistical significance calculated by Student’s t-test as indicated (***, *P*<0.001; **, *P*<0.01; *, *P*<0.05; ns, not significant).

Recombinant VACVs expressing GFP fused to the A5 capsid protein have been constructed in a wild-type (WT) background [5] and in viruses lacking either A36 (v∆A36) or F12 (v∆F12) expression [44]. To test if mutations in A36 and F12 have a cumulative effect on IEV egress or VACV spread, a virus lacking both genes, v∆A36∆F12 or v∆∆, was constructed. The parent virus used was v∆A36 A5-GFP and the *F12L* gene was disrupted by transient dominant selection (see Methods). During this process an intermediate virus is formed that contains the WT *F12L* gene and the deleted version. This unstable intermediate then resolves to either mutant or parental virus, which can be distinguished by PCR. Resolution of the intermediate virus gave two sizes of plaque and PCR analysis showed that all the larger plaques represented the v∆A36 A5-GFP parent, while all the smaller plaques were v∆A36∆F12. Single clones of a large (parental virus referred to as the sibling) and small plaque representing v∆A36∆F12 were plaque purified, amplified and their genotypes were analysed by PCR and confirmed to be as expected (Fig. 1a).

Plaque size analysis on BS-C-1 cells showed that both v∆A36 and v∆F12 produced plaques considerably smaller than WT virus with those formed by v∆F12 being significantly smaller than v∆A36 (Fig. 1b, c), as noted previously [45]. In comparison, v∆A36∆F12 produced plaques significantly smaller than v∆A36 and even smaller than v∆F12 (Fig. 1b, c). As expected, the v∆A36 sibling produced plaques indistinguishable from the parental v∆A36.

Plaque size can be affected by several factors including virus replication, egress, spread to new cells and blocking the host response to infection. To address if virus replication was altered, the yields of infectious intracellular virus were measured and showed no significant difference between the viruses (Fig. 1d). Next the egress of virus to the cell surface was measured by flow cytometry as previously described [43]. Unpermeabilized HeLa or U-2 OS cells were stained with an antibody to B5 to detect surface CEVs and then analysed by flow cytometry (Fig. 1e). Surface B5 levels were reduced in v∆A36-infected cells compared to WT virus infection and were reduced further in v∆F12-infected cells. There was no detectable difference in surface B5 staining between v∆A36∆F12 and v∆F12.

These results indicate that while deleting both F12 and A36 had a cumulative detrimental effect on virus spread, this was not due to a reduction in replication. The reduced efficiency of spread of vΔA36ΔF12 compared to vΔF12 was not a result of a reduction in virus transport to the cell surface. Therefore, it seems that the loss of the A36 protein is important for egress only if F12 is present and makes little difference if F12 is absent.

A36 contributes to VACV spread by two separate mechanisms. It links the IEV particle to kinesin-1 and it induces actin tail formation from the cell surface. Viruses lacking A36 have reduced virus egress and are unable to enhance spread by producing actin tails. Viruses lacking F12 show >99 % loss of IEV egress; however, the few virions that reach the cell surface can induce actin tail formation. Therefore, it is possible that the reason v∆F12 produces larger plaques than v∆A36∆F12 is the ability to produce a few actin tails. Alternatively, the difference may be due to small changes in egress caused by loss of A36 that were not detected by the egress assay shown in Fig. 1(e). To distinguish between these alternatives, additional viruses were constructed in which A36 was mutated to remove its ability to interact with kinesin, or to induce actin tail formation, or both activities. These mutations were introduced into viruses that did or did not express the F12 protein.

### Analysis of viruses with mutations in A36

**Fig. 2.**
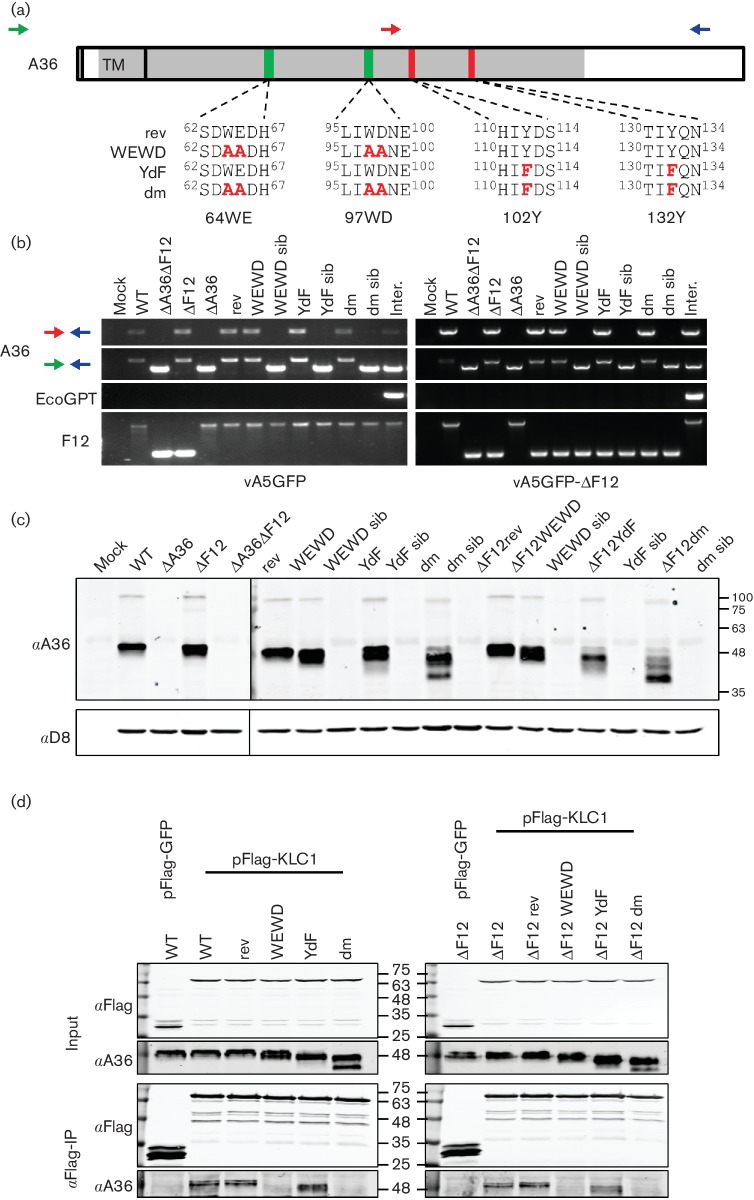
Construction and verification of a panel of viruses in which the two functions of A36 have been mutated individually. (a) Schematic representation of the A36 protein showing the localization of its transmembrane domain (TM), kinesin-interacting WE/D motifs (green) and tyrosines involved in nucleating actin polymerization (red). A panel of viruses was constructed by re-introducing full-length A36 into viruses in which a region of A36 had been deleted (shown in grey) to produce viruses with a WT A36 sequence (rev), with both WE/D motifs in A36 mutated to alanines (WEWD), with the actin nucleation tyrosines mutated to phenylalanine (YdF), or with both of these mutations to produce a double mutant (dm). Coloured arrows show position of primers used for PCR identification of recombinant viruses (see Methods). (b) PCR analysis of recombinant virus genomes. WT and mutant A36 genes were introduced into v∆A36-A5GFP as the parent (left panel) or v∆A36∆F12 (right panel). The presence of a full-length A36 allele in the recombinant viruses was confirmed using primers represented by the red and blue arrows in Fig. 2(a). No full-length A36 was detected in any of the isolated sibling viruses. Primers flanking the region deleted in ∆A36 viruses, represented by the green and blue arrows in Fig. 2(a), only detected the predicted genomes in the recombinant viruses. A PCR using primers specific for the EcoGPT selection cassette present in intermediate viruses did not detect this cassette in any of the purified virus stocks. A PCR that could distinguish between a full length *F12L* ORF and a deletion mutant was used to confirm the absence of any cross-contamination between the different recombinants. The identity of the mutant A36 alleles was also confirmed by sequence analysis. (c) A36 expression analysis by SDS-PAGE and immunoblotting. Monolayers of BS-C-1 cells were infected with the indicated viruses at 10 p.f.u. per cell. Lysates were harvested 16 h p.i., normalized by total protein concentration and analysed by immunoblotting. An antibody to the VACV D8 IMV surface protein was used as a loading control. The positions of molecular mass markers are shown (kDa). The image shown is representative of three experiments. (d) Co-immunoprecipitation analysis of A36/KLC interaction. HEK293T cells were transfected with a plasmid expressing Flag-KLC1 (or Flag-GFP as a negative control) and infected with the indicated viruses at 5 p.f.u. per cell for 14 h. Cell lysates were prepared and used for immunoprecipitation with anti-Flag beads (αFlag-IP). Input lysates (top) and immunoprecipitated proteins (bottom) were analysed by SDS-PAGE and immunoblotting. The positions of molecular mass markers are shown (kDa). The image shown is representative of two independent experiments.

The interaction of A36 with kinesin can be abrogated by mutating the two WE/D motifs found at positions 64 (WE) and 97 (WD) to alanines [35]. Similarly, the ability of A36 to induce actin tail formation can be abrogated by mutating tyrosines 102 and 132 to phenylalanine [46]. Recombinant viruses were generated by re-introducing either WT or mutant A36 alleles into ∆A36. Re-introduction of WT A36 generated a revertant virus referred to as rev. Insertion of A36 incorporating the 64WE and 97WD into alanine mutations formed a virus named WEWD. Insertion of A36 bearing the Y102F and Y132F mutations formed a virus called YdF, and insertion of A36 bearing both WEWD and YdF mutations produced a virus called double mutant or dm (Fig. 2a). One set of viruses was generated using v∆A36-A5GFP as the parental strain and another set was generated using v∆A36∆F12-A5GFP, described above, to generate the same panel of A36 mutants in a background lacking F12 expression. Both recombinant and parental sibling clones were plaque purified for each of the mutant viruses. Clonal identity and purity were verified by PCR (Fig. 2b) and sequence analysis of the A36 locus.

Immunoblot analysis of A36 expressed by the various recombinant viruses showed that there were some changes in electrophoretic mobility on SDS-PAGE and in particular the dm A36 protein had several different forms (Fig. 2c). Interestingly, the banding pattern of A36 was altered by the presence or absence of F12. The ability of these mutant A36 proteins to interact with KLC was examined by immunoprecipitation. Cells were transfected with Flag-tagged KLC (isoform 1) and then infected with the different viruses. As expected, viruses expressing A36 with intact WE/D motifs (rev and YdF mutants) co-immunoprecipitated with KLC1, while those in which the WE/D motifs had been mutated (WEWD and dm) did not (Fig. 2d).

### A36-mediated kinesin-1 recruitment can contribute to virus egress only in the presence of F12

**Fig. 3.**
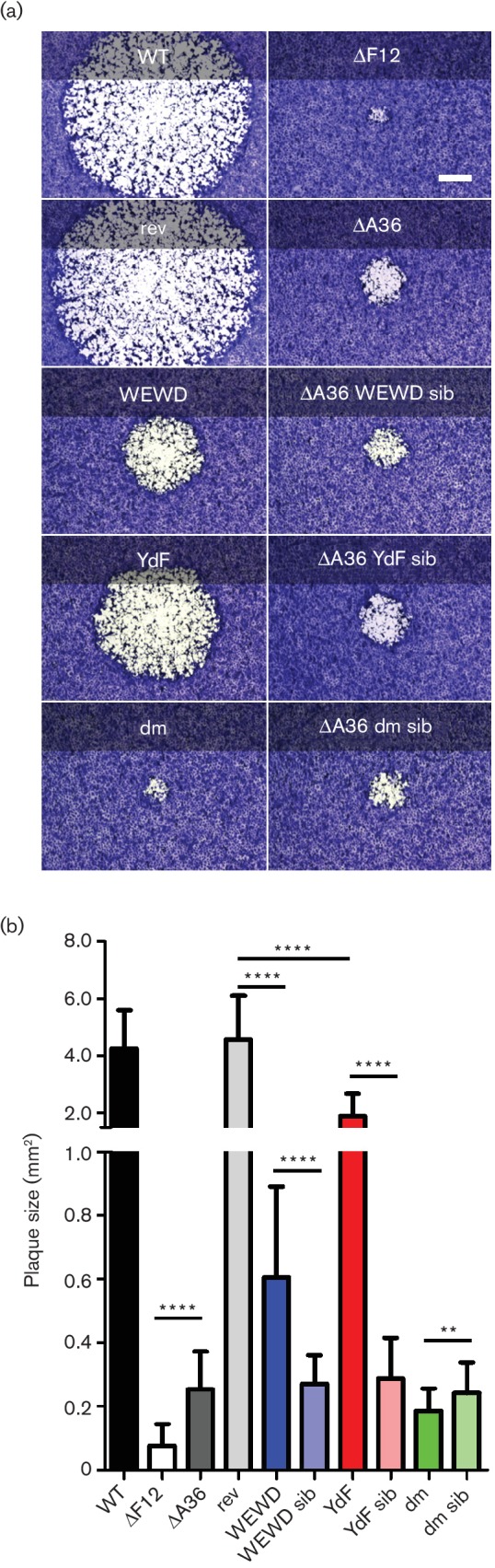
Plaque size comparison of viruses expressing F12. (a) Plaque size comparison of the indicated recombinant viruses 3 days p.i. Images of single representative plaques are shown; bar, 500 µm. (b) Quantitative analysis of plaque sizes. A minimum of 15 plaques from three independent infections were measured (total of 45 plaques). Graph shows mean plaque area ±sd. Mutant A36-expressing viruses were compared statistically to the WT A36-expressing rev virus and/or to their sibling virus as indicated using Student's t-test (****, *P*<0.0001; **, *P*<0.01).

To analyse the contribution of each of the functions of A36 to virus spread, the plaque size of each mutant virus was analysed on BS-C-1 cells and compared to viruses lacking F12, or A36, or both proteins (Fig. 3). Re-introduction of WT A36 into v∆A36 rescued the plaque size to WT, while all the mutant A36-expressing viruses produced plaque sizes smaller than WT. The YdF virus produced plaques almost half the area of WT plaques and the WEWD virus plaques were about a quarter the area of WT. If kinesin-1 binding and actin nucleation are the only functions of A36 then knocking both of these out should result in a plaque size similar to v∆A36. Instead the dm virus produced plaques even smaller than v∆A36, suggesting that the presence of the dm A36 can interfere with virus spread. The observation that both YdF and WEWD mutants produced larger plaques than dm or v∆A36 indicated that, in a WT background, both kinesin-1 interaction and actin polymerization contribute to virus spread.

**Fig. 4.**
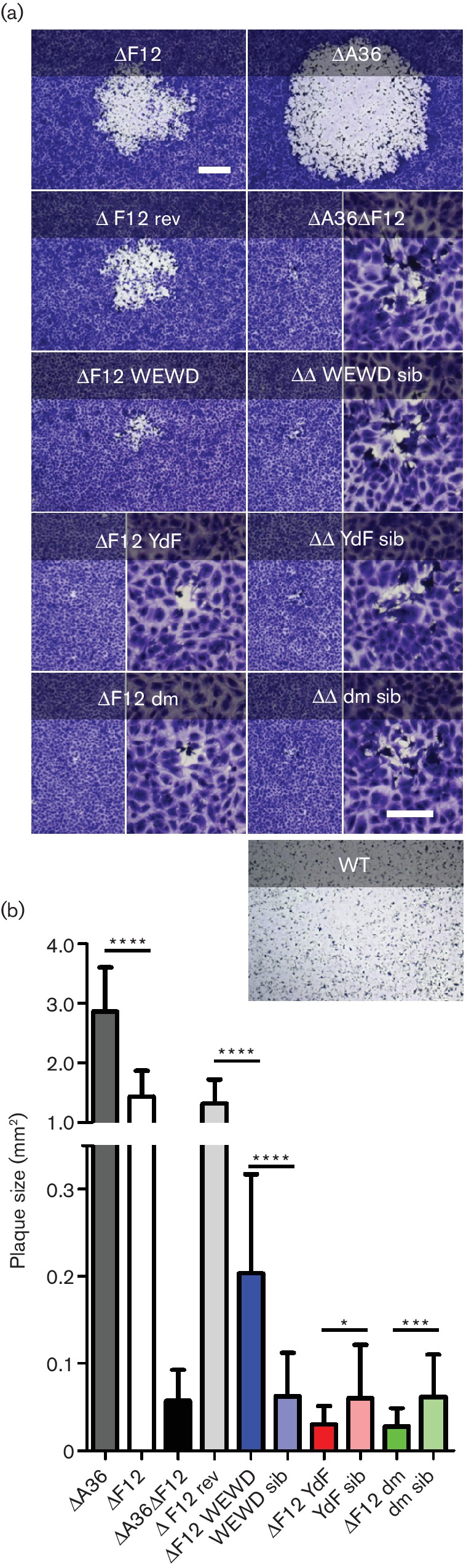
Plaque size comparison of viruses in the absence of F12 expression. (a) Plaque size comparison of indicated viruses 9 days p.i. Images of single representative plaques are shown; bar, 500 µm. Higher magnification images are provided for the smallest plaques (inserts) with a scale bar of 200 µm. (b) Quantitative analysis of plaque sizes. A minimum of 45 plaques from three independent infections were measured. Graph shows mean area ±sd. Mutant A36-expressing viruses were compared statistically to the WT A36-expressing rev virus and/or to their sibling virus as indicated using Student's t-test (****, *P*<0.0001; ***, *P*<0.001; *, *P*<0.05).

The contribution of these mutant A36 proteins was studied next in the ∆F12 background (Fig. 4). Re-introducing WT A36 rescued the plaque size back to that of v∆F12. However, of the mutant A36 alleles, only the WEWD virus rescued plaque size at all, and only to about one-fifth of the area of plaques produced by v∆F12 rev. Notably, unlike in the WT background expressing F12, in the absence of F12 the YdF A36 mutant was unable to rescue plaque size. In fact, both YdF and dm viruses produced plaques smaller than the v∆A36∆F12 parent or sibling viruses. These results indicated that in the absence of F12, the ability of A36 to interact with kinesin-1 is unable to contribute to viral spread.

**Fig. 5.**
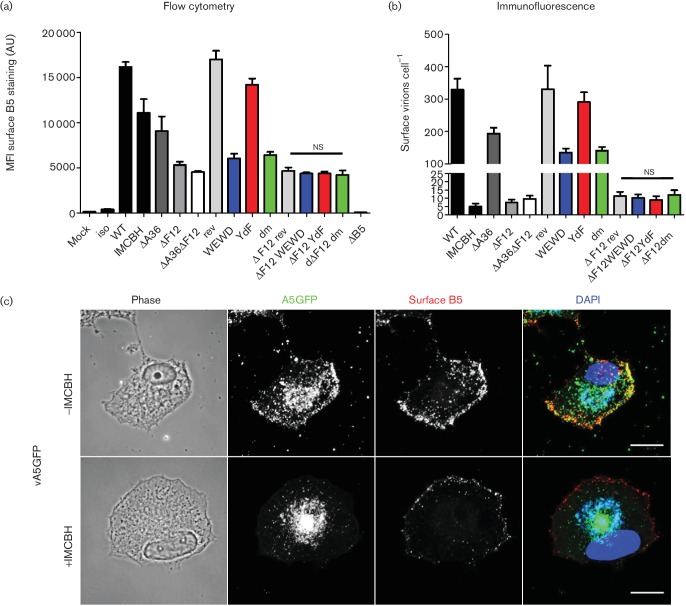
Quantitation of IEV egress. Virion egress was measured by live cell surface staining of protein B5. Levels of staining were quantified by two alternate methods. (a) Flow cytometric quantitation of infected cells surface stained for B5 and fixed at 12 h p.i. The graph shows the mean measurements of three independently-infected samples. Values represent the mean fluorescence intensity (MFI), given in arbitrary units (AU) per cell, of a minimum of 2000 events per replicate. Error bars represent standard error of the mean (sem). Cells infected with WT virus but stained with an isotype control antibody (iso), and cells infected with v∆B5 but stained with an anti-B5 antibody are included as controls. Infection with WT virus in the presence of a drug that inhibits IEV wrapping (IMCBH) is also included. Results shown are representative of three independent experiments. (b) Surface virion quantitation by immunofluorescence confocal microscopy of cells infected with the recombinant viruses and stained for surface B5 at 8 h p.i. Confocal *z*-stacks of the entire cell volume were acquired and surface virions (positive for GFP and B5) were counted manually. Values represent means of a minimum of 12 cells per sample with error bars representing sem. For (a) and (b), egress levels of samples ∆F12rev, ∆F12WEWD, ∆F12YdF and ∆F12dm were compared by one-way ANOVA and found to not be significantly different (ns), nor did they differ significantly from ∆F12 or ∆A36∆F12. The difference between rev and YdF was tested by Student's *t*-test and found not to be statistically different, nor did WEWD differ significantly from dm. (c) Immunofluorescence of BS-C-1 cells infected with vA5GFP and stained for surface B5 8 h p.i. ±IMCBH. Bars, 20 µm.

To measure the efficiency of egress of the different mutant viruses, the egress assay described above (Fig. 1e) was used again (Fig. 5). In this case, egress was not only measured by flow cytometry (measuring the total cell surface B5 levels, Fig. 5a), but also by immunofluorescence microscopy to quantify B5-positive GFP puncta (virions) on the cell surface (Fig. 5b) giving a direct measurement of the number of virions that reach the cell surface (described in Methods and in [44]).

The YdF mutation should theoretically have no effect on egress compared to the rev virus; however, both assays show a small drop in the level of cell surface virions. Likewise the WEWD mutant would be expected to reduce egress to the level of v∆A36. Instead, it seems to reduce egress slightly further. The levels of egress of the WEWD and dm mutant viruses were indistinguishable in both assays, indicating that the plaque size difference shown in Fig. 3 is attributable to the ability of WEWD to form actin tails. However, for the viruses made in the ∆F12 background it did not matter what version of A36 was present. The levels of egress by v∆F12 and v∆A36∆F12 were indistinguishable. Therefore, in the absence of F12, the ability of A36 to engage kinesin-1 is unable to contribute to egress.

**Fig. 6.**
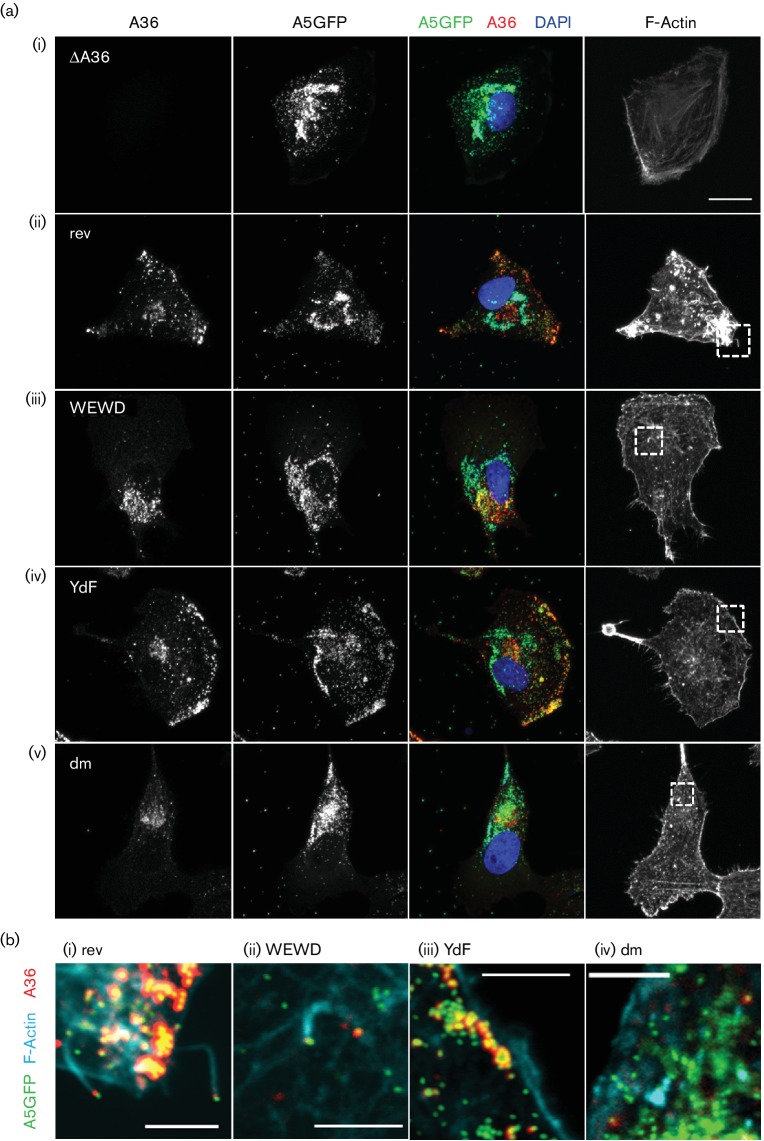
Immunofluorescence analysis of infected cells. BS-C-1 cells infected with the indicated viruses at 5 p.f.u. per cell were fixed 8 h p.i. and stained for the presence of A36 and filamentous actin. (a) The A36 staining (red) and GFP signals (green) are shown as monochrome images and as an RGB (red-green-blue) merge together with DAPI (blue). The F-actin signal is represented as a separate monochrome image. All images are maximum intensity projections of a *z-*stack encompassing the full volume of the cell imaged and are represented at the same magnification. Bar, 20 µm. (b) The boxed regions in (a) are presented as a single optical section at a higher magnification to show A36 (red) and A5GFP (green) co-localization and F-actin (cyan) showing presence or absence of actin tails. Bars, 5 µm. The absence of actin tails was confirmed for the YdF and dm mutants after looking at 75 infected cells for each virus.

**Fig. 7.**
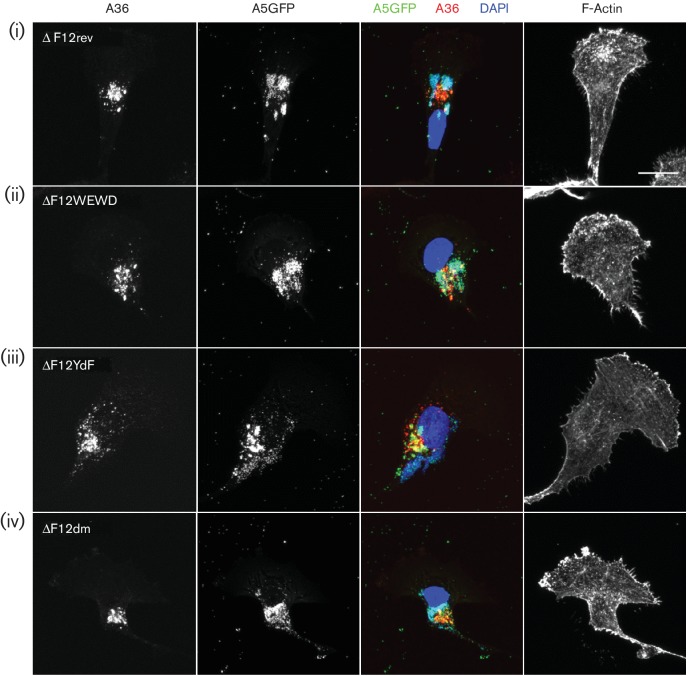
Immunofluorescence analysis of cells infected with viruses lacking F12. BS-C-1 cells infected with the indicated viruses and processed as in Fig. 6(a). Bar, 20 µm.

As a side note, in the egress assays a sample was included as a negative control in which cells infected with vA5GFP were treated with the drug IMCBH (*N1*-isonicotinoyl-*N2*-3-methyl-4-chlorobenzoylhydrazine), an inhibitor of poxvirus spread that binds the F13 protein and interferes with the wrapping of IMV to form IEV [47, 48]. In the presence of IMCBH, the lack of IEV formation should result in a loss of virus egress. However in the flow cytometry assay, IMCBH-treated cells showed high levels of surface B5 staining (Fig. 5a) suggesting egress was taking place. When the assay was repeated by immunofluorescence, IMCBH-treated cells were found to have high levels of surface B5 staining (Fig. 5a, c) but practically none of this surface B5 co-localized with virion GFP signal (Fig. 5b, c). Lastly, immunofluorescence confocal microscopy of infected BS-C-1 cells was used to assess the subcellular distribution of the A36 mutant proteins and their ability to induce actin tail formation (Figs 6 and 7). A36 accumulates at a perinuclear site representing the site of IMV wrapping which is near to, but separate from, virus factories. All of the mutant A36 proteins showed this localization whether F12 was present (Fig. 6) or absent (Fig. 7). A36 proteins capable of binding kinesin-1, in the presence of F12, showed secondary accumulation at the cell periphery (Fig. 6a) co-localizing with virions [Fig. 6b(i), b(ii)]. However, when A36-mediated recruitment of kinesin-1 was abrogated, either because A36 was absent, or because the WE/D motifs were mutated, virions did not localize to the cell periphery and instead accumulated at the site of wrapping (Fig. 6a). In the absence of F12, this accumulation was evident for both WT and all mutant versions of A36 (Fig. 7). In the presence of F12, actin tails were present when WT A36 was expressed but absent for the YdF and dm mutants lacking the actin nucleation motifs [Fig. 6b(iii), b(iv)]. Actin tails were more difficult to find for the WEWD mutant than for the rev virus, reflecting the reduction in virus egress. When actin tails were present they often formed, not at the cell periphery, but at the apical or basal cell surface nearer to the cell nucleus and the site of wrapping [Fig. 6b(ii)].

## Discussion

In the absence of A36, IEVs are still transported on MTs but the run length is reduced and, although there is a delay in their transport, surface virions reach 50 % of WT level by 24 h post-infection [44]. In the absence of F12, there is a much more severe reduction in egress to the cell periphery and surface virions represent less than 1 % of WT levels [26, 44]. This is surprising given that A36 links IEVs directly to kinesin [31] and is required for F12 to associate with IEVs [42]. To analyse the relative contributions of A36 and F12, a virus defective in the expression of both proteins was constructed. As would be expected if A36 and F12 mediated egress by different pathways, this virus produced plaques smaller than viruses lacking A36 or F12 expression individually. However, the contribution of A36-mediated actin tail formation to virus spread, and consequently to plaque size, complicated interpretation of this observation. Additionally, actin tails enhance the rate of virus spread by repelling superinfecting virions [40]. Measuring the level of virion egress by staining surface virions with antibody to B5 suggested there was no difference in egress efficiency between v∆A36∆F12 and v∆F12. Actin tails can also enhance virion release from the cell surface into the extracellular medium [49]. This has the potential to skew or mask any differences in surface virions when comparing a virus lacking actin tails with one that can produce actin tails.

**Fig. 8.**
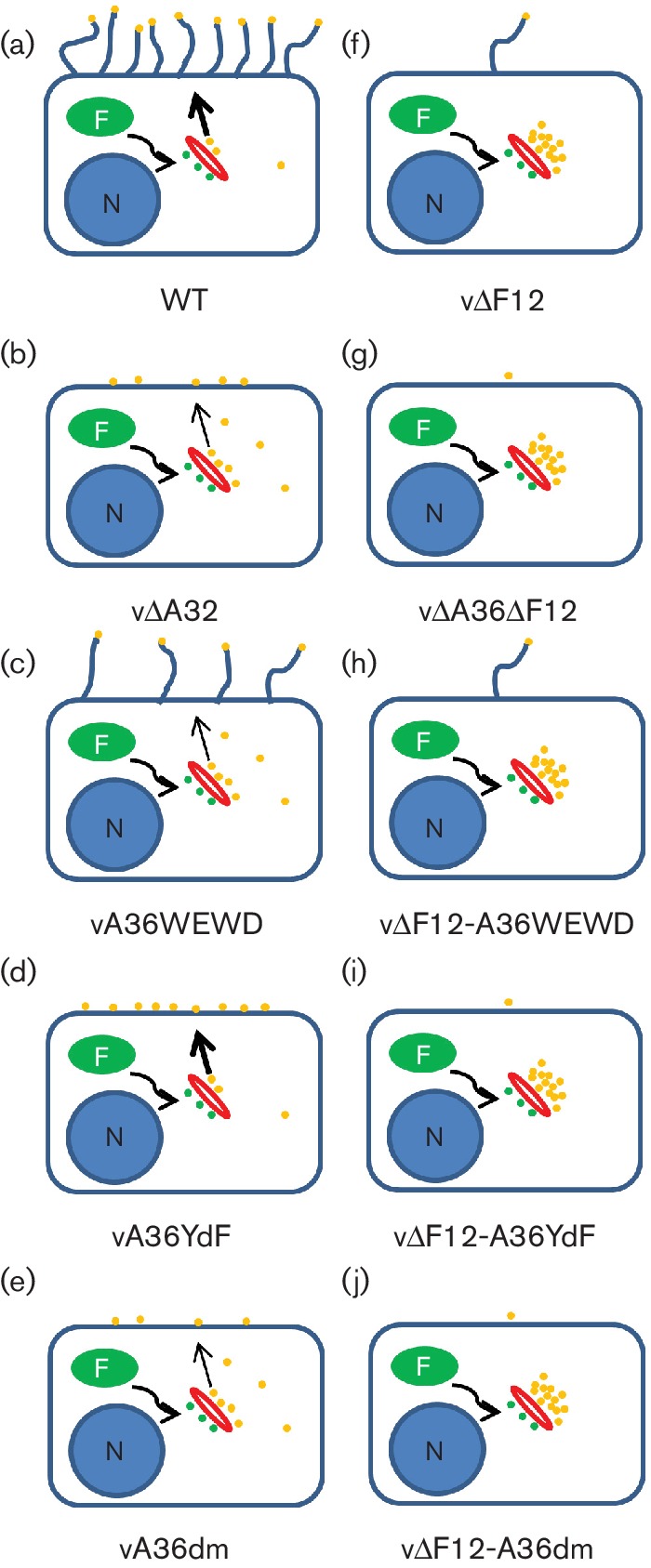
Schematic summary of recombinant mutant virus phenotypes. Schematic representation of the relative efficiency of virus egress and spread events in the different mutant viruses analysed. Virus replication and assembly takes place in cytoplasmic structures referred to as viral factories (F) leading to the formation of IMVs. Some IMVs (green virions) migrate away from the viral factories and are wrapped to become IEVs (yellow virions), which are transported to the cell periphery. Virions on the cell surface (CEVs) can induce the formation of actin tails. In cells infected by viruses with a WT genotype (including vA5GFP and vA36rev-A5GFP) levels of egress are normal and virions at the cell surface can form actin tails (a). Viruses lacking A36 have a reduced efficiency of virion egress, resulting in some virions accumulating at the site of wrapping, and those virions that do make it to the cell surface are unable to form actin tails (b). Restoring A36 expression with a mutant unable to bind to KLC (vA36WEWD-A5GFP) restores the ability to form actin tails but not normal levels of virus egress (c). If A36 expression is restored with a mutant unable to nucleate actin polymerization, virion egress is restored to levels similar to viruses expressing WT A36, but no actin tails are formed (d). Viruses expressing A36 in which both functions have been mutated show egress levels similar to A36WEWD-expressing viruses but are unable to form actin tails (e). Viruses lacking F12 have drastically reduced IEV egress, resulting in accumulation of IEVs at the site of wrapping; however, those virions that reach the cell surface are able to form actin tails (f). Disrupting A36 expression in these viruses does not alter the level of IEV egress but prevents actin tail formation, resulting in the plaque size reduction described in Fig. 1(g). All of the mutant A36-expressing viruses in which F12 expression has been disrupted show egress levels identical to WT A36-expressing viruses, irrespective of whether A36 can interact with KLC. Introducing an A36 that can induce actin polymerization (h) rescues plaque size slightly (though to levels below that of WT A36-expressing viruses). Those viruses unable to form actin tails produce plaque sizes slightly smaller than viruses that lack A36 altogether (Fig. 4) irrespective of whether A36 can (i) or cannot (j) interact with KLC unlike for the viruses expressing F12 where the ability of A36 to bind KLC leads to enhanced plaque size (Fig. 3). N, Nucleus.

By constructing a panel of viruses in which A36-mediated actin polymerization was abrogated, either alone or in concert with disruption of the A36/KLC interaction, the confounding factor of actin tails was eliminated. Comparison of viruses expressing A36 capable of KLC recruitment with viruses expressing A36 unable to bind KLC (comparing YdF to dm) revealed that, in the presence of F12, A36 kinesin-1 recruitment resulted in increased egress. In contrast, in the absence of F12 it does not (see Fig. 8 for a summary of the phenotypes of all the viruses analysed). A36-mediated egress is thus completely reliant on F12. It has been reported that F12 is required for IEV formation rather than egress [28]. However, electron microscopy evidence of fully formed IEVs in the absence of F12 and the observation that IEVs are more abundant in infections with viruses lacking F12 than during WT VACV infection [33] refute this. Similarly, loss of A36 does not affect IEV formation [50, 51]. Therefore, plaque size reduction and reduced virus egress resulting from disruption of either F12 or A36 are due to defects downstream of IEV formation.

Unlike F12 and A36, F13 is required for IEV wrapping and this is inhibited by IMCBH [47, 48]. Therefore, IMCBH was used to measure baseline B5 levels when wrapping and, consequently, egress are inhibited. This showed that, while surface virions are virtually absent after IMCBH treatment, B5 is present on the cell surface. This is reminiscent of the recent observations made by Sivan *et al.* [52] when cellular retrograde trafficking was disrupted. F13 and B5 are both required for IEV wrapping [53] and both localize to the membranes that form the IEV envelopes. However, these proteins take alternative routes to reach the membranes at the site of wrapping [52]. There is some evidence that B5 reaching the plasma membrane is re-internalized by endocytosis [54] similar to the mechanism by which herpes simplex virus glycoproteins are transported to the membranes used to form the virion envelope [55]. It is unclear whether the presence of IMCBH enhances B5 transport to the cell surface, or if B5 recycling by endocytosis is somehow inhibited by IMCBH.

In the absence of A36, some IEV egress on MTs still occurs [44]. Yet A36 is the only VACV protein known to form a direct link between IEVs and kinesin-1 [31]. This suggests that another link between IEVs and kinesin-1 exists, yet none of the other viral IEV-associated proteins tested interacts with kinesin [31]. The membranes that form the IEV envelopes contain cellular proteins [56], and being of endosomal or trans-Golgi origin, it is likely that some of these proteins can recruit motor proteins, including kinesin-1 [57]. Any protein possessing WE/D motifs can complement the loss of A36-mediated recruitment of kinesin-1 [35]. Unlike F12, which is conserved in all known vertebrate poxviruses (*Chordopoxvirinae*), A36 orthologues are less conserved outside of the orthopoxviruses. Viruses from several other chordopoxvirus genera, including the yatapoxviruses (Yaba-like disease virus [58]), avipoxviruses (fowlpox virus [59]) and leporipoxviruses (myxoma virus [60]) have been reported to induce actin tails. In some cases, A36 orthologues capable of inducing actin tails were identified despite low sequence identity (9–14 %) [61]. Putative WE/D motifs were identified in these non-orthopoxvirus A36 orthologues [61], but their ability to recruit kinesin-1 remains to be tested.

F12, as part of the F12/E2 complex [28], can interact with KLC in the absence of A36 [43]. This interaction may induce an allosteric shift that primes the kinesin-1 complex for binding to other cargo proteins (such as A36) or for activating its motor activity. VACV and other poxviruses are therefore flexible in the mediator they use to link IEVs to kinesin-1. However, in the absence of a functional F12/E2 complex this does not lead to virus egress. That F12 can contribute to egress in the absence of A36 is clear, both from data presented here and from past observations. F12 lacking a region that has been mapped as being involved in its interaction with A36 does not associate with IEVs [42]. It is unclear however if this mutant F12 is still functional in other respects. Both F12 and E2 require the other to be present to associate with IEVs [28]. Whether full-length F12 or E2 can associate with IEVs in the absence of A36 remains to be determined.

In summary, a mutant virus lacking both F12 and A36 forms a smaller plaque than viruses lacking either protein alone, showing non-redundant properties. The ability of the A36 protein to bind kinesin-1 and nucleate actin tails both contribute to virus spread when protein F12 is present, but in the absence of the F12 protein whether or not A36 can bind KLC makes no difference to egress and spread, showing reliance on the F12 protein.

## Methods

### Cells

RK-13 cells (rabbit kidney cell line, ATCC CCL-37) were maintained in minimal essential medium (Gibco) supplemented with 10 % foetal bovine serum (FBS) and penicillin-streptomycin (Pen/Strep, Gibco). HeLa cells (human cervix adenocarcinoma, ATCC CCL-2) were maintained in minimal essential medium supplemented with 10 % FBS, and non-essential amino acids and Pen/Strep (Gibco 11140). HEK 293T (human embryonic kidney cell line, ATCC CRL-11268), U-2 OS (human osteosarcoma cell line, ATCC HTB-96), CV-1 (African green monkey cell line, ATCC CCL70) and BS-C-1 (African green monkey cell line, ATCC CCL-26) cell lines were maintained in Dulbecco's modified Eagle medium (Gibco) supplemented with 10 % FBS and Pen/Strep.

### Construction of recombinant viruses

All viruses used in this study expressed the A5-GFP fusion protein, which was introduced into a WT VACV Western Reserve strain [5] and its ∆F12 and ∆A36 mutants [44]. A v∆F12 was constructed by introducing a 1207 bp internal deletion [26] but for the purposes of this study another v∆F12 was constructed that deleted more of the F12 ORF. A *Bam*HI fragment was subcloned from p∆F12 [26] into the *Bam*HI site of pUC13-EcoGPT-mCherry [43]. A DNA fragment encompassing the last 43 bp of the *F12L* ORF and 365 bp downstream from VACV WR was generated by PCR using oligonucleotides F12-DnStrF1 (5′-CACCCCCGGGTAAAGATATTAATGAA-TCCATGAGTCAGATG-3′) and F12-DnStrR1 (5′-CACCGAATTCCAGCATCTAACTTGATGTCAGG-3′) and cloned into the *Xma*I-*Eco*RI sites of the same plasmid to generate pEcoGPT-mCherry-∆F12 mkII. This plasmid was used to generate the v∆A36∆F12-A5GFP virus by transient dominant selection [62, 63]. Briefly, CV-1 cells were transfected with the plasmid and infected with the parent virus. Recombinant viruses that incorporated the plasmid by recombination and expressed the EcoGPT-mCherry fusion protein were selected by serial passage and plaque purification on monolayers of BS-C-1 cells under selection by mycophenolic acid supplemented with hypoxanthine and xanthine. After isolation of an intermediate clone, the virus was plaque purified three additional times without selection to isolate clones that had undergone a second recombination event and lost expression of EcoGPT-mCherry. Viruses that had incorporated the new ∆F12 mkII allele were differentiated from those that had reverted to a parental genotype by PCR and sequence analysis. The virus lacking both F12 and A36 (double deletion virus) was constructed by using v∆A36-A5GFP as parental virus. In parallel, the ∆F12 mkII allele was used to replace the ∆F12 allele in v∆F12-A5GFP to generate viruses with identical deletions at the F12 locus. Analysis of this new v∆F12, used for the rest of this study, confirmed it had the same phenotype as the original parental virus.

The pEcoGPT-mCherry-A36rev plasmid was generated by amplifying the A36 locus by PCR, including about 350 bp upstream and downstream of the ORF, using oligonucleotides A36 UP F (5′-GATCAAGCTTTGGCTAGATTCAACATT TATAGCATTTGTG-3′) and A36 DN R (5′-GA-TCGAATT CACGACACATTTACTCAGTGGGGATATG-3′) and cloning the product into the *Hin*dIII-*Eco*RI sites of pUC13-EcoGPT-mCherry. The pEcoGPT-mCherry-A36YdF plasmid was generated by amplifying two DNA fragments by PCR using VACV WR genomic DNA template and oligonucleotides A36 UP F with A36YdF R (5′-ACTGTAG-TGTTCTGAAAAATAGTCTGTTCATTACGATCATTATTTATTA GCAGCGTGCTTCCAGCAACACTATCGAA-3′), and A36 DN R with A36YdF F (5′-AGCACAGAACACATTTTCGATAGTGTTGCTGGAAGCACGCTGC-TAATAAATAATG ATCGTAATGAACAGACTATTTTTC-3′). The two fragments incorporating the YdF mutations were spliced by overlap extension and inserted into the *Hin*dIII-*Eco*RI sites of pUC13-EcoGPT-mCherry. The pEcoGPT-mCherry-A36WEWD and pEcoGPT-mCherry-A36dm plasmids were generated by splicing two DNA fragments generated by PCR using pEcoGPT-mCherry-A36rev as template and oligonucleotides A36atgKpnIF (5′-ATGATGCTGGTACCTCTTATCAC-3′) with A36NarI-WD/AA R (5′-GATGGGCGCCATGACATTGGATTCG-TTAGCCGCTATTAAACTACC-3′) and A36-WE/AA SOE F (5′-TCAGACGCTGCAGATCACTGTAGTGCTATGGAAC-3′) with A36-WE/AA SOE R (5′-GTGATC-TGCAGCGTCTGATTCGCTATCAGTTGATTT AC-3′) and inserting the product into pEcoGPT-mCherry-A36rev and pEcoGPT-mCherry-A36YdF, respectively.

These plasmids were all used to generate recombinant viruses as described above using v∆A36-A5GFP as the parental virus to generate recombinants expressing both F12 and a mutant form of A36, and v∆A36∆F12-A5GFP as the parental virus to generate recombinants expressing the mutant A36 proteins but lacking F12 expression.

Viruses were analysed by PCR to identify recombinants. PCRs were used to distinguish the WT F12 and ∆F12 alleles (oligonucleotides F12RevDC1 5′-CATCTTTGATCTCGA-TGGAATGCA-3′ with F12ForDC1 5′-ATGTTAAACA-GGGTACAAATCTTGATGA-3′), the presence or absence of the EcoGPT cassette (oligonucleotides EcogptDCF1 5′-CGTCACCTGGGACATGTTG-3′ with EcogptDCR1 5′-GACGAATACGACGCCCATAT C-3′), the full length and ∆A36 alleles [oligonucleotides A36 UP F see above (green arrow in Fig. 2a) with DCA36seqR1 5′-ACCGTTTCAT-CCATCTGTCTATTG-3′ (blue arrow in Fig. 2a)], and the full length A36 using oligonucleotide A36deltestF (5′-CTTA-TCACGGTGACCGTAG-3′, red arrow in Fig. 2a) with DCA36seqR1. To verify that the correct A36 mutations had been incorporated into the final recombinant viruses PCR fragments were sequenced.

### Plaque size analysis

Monolayers of BS-C-1 cells in six-well plates were infected with 20–30 p.f.u. per well. Plaques were left to develop for the indicated time under a semi-solid overlay containing 1.5 % carboxymethyl-cellulose. Plaques were either stained with crystal violet or fixed with 4 % paraformaldehyde in PBS for 30 min at room temperature. Plaques stained with crystal violet or GFP-positive plaques were imaged using an Axio Observer.Z1 inverted microscope. Historically we have estimated plaque size by drawing a circle around it, using tools available in the AxioVision (Zeiss) imaging software package, and determining the radius. Plaques are however not perfect circles. In our experience this approach is sensitive enough to detect moderate to large plaque size changes but fails to detect small changes in plaque size. During this study we shifted from measuring plaque radius to the more accurate approach of measuring the exact surface area of individual plaques using the spline selection tool available in AxioVision (release 4.8). While initial measurements comparing v∆A36, v∆F12 and v∆A36∆F12 were done using the original method (Fig. 1), measurements for these viruses were repeated using surface area measurements (Figs 3 and 4). For each sample, a minimum of 15 measurements from three independent wells (for a total of 45 measurements) were used to carry out statistical analysis using the statistical software package GraphPad Prism (version 5.04).

### Immunofluorescence microscopy

BS-C-1 cells were seeded onto poly-d-lysine-coated glass coverslips (19 mm diameter, thickness no.1) in 12-well plates and left to attach for a minimum of 36 h prior to infection. Cells were infected with 5–10 p.f.u. per cell and fixed 8 h post-infection (p.i.) with 4 % paraformaldehyde in 250 mM HEPES pH 7.5 for 30 min on ice followed by 8 % paraformaldehyde in 250 mM HEPES pH 7.5 for 15 min at room temperature.

Fixed cells were blocked with 10 % FBS and 5 % goat serum (D9663; Sigma) in PBS and permeabilized with 0.1 % Triton X-100. Cells were stained with anti-A36 mouse monoclonal antibody [23], Alexa Fluor 546-conjugated donkey anti-mouse secondary antibodies (Life Technologies) and Alexa Fluor 647-conjugated phalloidin. Cells were mounted [10 % (w/v) Mowiol 4-88 (CalBiochem), 25 % (v/v) glycerol, 100 mM Tris-HCl pH 8.5, 0.5 µg ml^−1^ DAPI (4′,6-diamidino-2-phenylindole; Sigma)] and imaged using a Zeiss LSM780 confocal laser scanning microscopy system mounted on an AxioObserver.Z1 inverted microscope using a ×64 Plan Apochromat objective (numerical aperture 1.4) and Zen (Zeiss, 2011 version) acquisition software. z-Stacks encompassing the full volume of the cell were acquired and used to generate maximum fluorescence intensity projections down the *z*-axis. Images were processed and analysed using Zen, ImageJ and Photoshop (Adobe) software.

### IEV egress assay

For flow cytometric analysis of surface B5 levels, BS-C-1, HeLa or U-2 OS cells were seeded into 24-well plates and left to settle for at least 36 h prior to infection. Cells were infected at 10 p.f.u. per cell. At 12–16 h p.i., cells were placed on ice and stained for B5 as previously described [43]. Fluorescence was measured using a Becton Dickinson Cytek FACScan DxP8 and analysed using MoFlo software. Statistical analysis of triplicate samples was carried out using GraphPad Prism statistical analysis software.

For analysis of virus egress by immunofluorescence, BS-C-1 cells were seeded onto poly-d-lysine-coated coverslips and infected as described above. At 8 h p.i., cells were placed on ice and stained for B5 as for flow cytometric analysis except that cells were fixed after the primary antibody as described above and stained with an Alexa Fluor 647 goat anti-rat secondary antibody (Life Technologies). The cells were imaged as described above and surface B5-positive, GFP-positive virions were counted manually using ImageJ software.

The drug *N1*-isonicotinoyl-*N2-*3-methyl-4-chlorobenzoylhydrazine (IMCBH) was used in several of our assays as an inhibitor of VACV wrapping. IMCBH was provided by R. Wittek from the University of Lausanne (Lausanne, Switzerland). IMCBH was used at a final concentration of 5 µg ml^−1^.

### Co-immunoprecipitation and immunoblot protein analysis

Co-immunoprecipitation analysis was carried out as described [43]. Briefly, cells in 10 cm diameter dishes were transfected with Flag-KLC1- or Flag-GFP- expressing plasmids using transit-LT1 transfection reagent (Mirius) and 24–36 h later were infected at 5 p.f.u. per cell for 14 h. Cells were then lysed in IP wash buffer [50 mM Tris-HCl pH 7.5, 0.5 % (v/v) Nonidet P40 substitute (Sigma), 150 mM NaCl, 2 mM EDTA] supplemented with complete Mini EDTA-free protease inhibitor cocktail tablets (Roche), on ice for 45 min with occasional vortexing. Lysates were clarified by centrifugation (15 000 ***g***, 15 min, 4 °C) and Flag-tagged proteins were immunoprecipitated using anti-FLAG M2 affinity gel (Sigma). Immunoprecipitations were incubated with rotation overnight and then washed four times with IP wash buffer. Immunoprecipitated proteins were eluted by boiling in Laemmli loading buffer and analysed by SDS-PAGE and immunoblotting.

Proteins separated by SDS-PAGE and transferred onto Hybond ECL nitrocellulose membrane (GE Healthcare) were probed with rabbit polyclonal αFLAG (F7425; Sigma-Aldrich, 1 : 5 000), mouse monoclonal αA36 [23] and mouse monoclonal AB1.1 specific for the VACV protein D8 [22]. Blots were probed with IRDye-conjugated secondary antibodies (LI-COR), and imaged using a LI-COR Odyssey scanner. Detection of the A36 protein, which has a similar molecular mass to the antibody heavy chain, was achieved by using a Biotin-SP AffiniPure goat anti-mouse IgG, light chain specific (115-065-174; Jackson ImmunoResearch). Biotinylated secondary antibodies were visualized using IRDye-conjugated streptavidin (LI-COR).
